# Circadian rhythm disturbances in mood disorders: characterisation and clinical impact

**DOI:** 10.1192/j.eurpsy.2023.464

**Published:** 2023-07-19

**Authors:** G. Cappannini, S. Bianchi, G. Menculini, B. Semeraro, F. De Giorgi, K. Amantini, P. Moretti, A. A. V. Tortorella

**Affiliations:** ^1^Department of Psychiatry; ^2^School of Medicine, University of Perugia; ^3^Section of Psychiatry, Clinical Psychology and Rehabilitation, Santa Maria Della Misericordia Hospital; ^4^Psichiatric Inpatient Unit Department of Mental Health, AUSL Umbria 1, Perugia, Italy

## Abstract

**Introduction:**

Circadian rhythms, defined as endogenous oscillations that regulate metabolism, physiology and behaviour, may be frequently disrupted in mood disorders, influencing their clinical presentation and course (Srinivasan V. *et al.* World J Biol Psychiatry. 2006;7(3):138-151).

**Objectives:**

To characterise circadian rhythm disruptions in a population of patients with mood disorders, analysing clinical and course differences in subjects with and without clinically significant circadian rhythm alterations

**Methods:**

Patients selected for this cross-sectional study were assessed with CGI-BP, HAM-D, MRS, and PANSS. Circadian rhythm disturbances were evaluated with BRIAN. Patients with clinically relevant circadian rhythm disturbances were defined as BRIAN > 36 (Mondin TC *et al.* J Psychiatr Res. 2017;84:98-104). Bivariate analyses were subsequently performed to compare subgroups of patients.

**Results:**

In our study, 61 subjects with DD or DB were enrolled. The overall mean BRIAN test score was 40.08 ± 10.26. When comparing the BRIAN test scores, both total and subscales, between subjects with DB and DD, social rhythms were significantly more altered in subjects with DB (8.63 ± 2.90 VS 6.80 ± 2.11, p=0.034). Subjects with disruption of circadian rhythms displayed greater severity of depressive symptoms (mean total HAM-D test score 16.06±8.61 VS 8.94±5.85; p<0.003, mean CGI-BP severity of depression test score 3.14±1.68 VS 1.88±1.11; p<0.010) and with a longer duration of untreated illness (6.14±8.64 VS 2.53±6.28; p= 0.040).

**Conclusions:**

Alterations in circadian rhythms should be routinely investigated in all individuals with mood disorders, especially BD, and may represent a transdiagnostic psychopathological construct that defines a more severe disease phenotype.

**Disclosure of Interest:**

None Declared

